# Temporal relationship between triglyceride-glucose index and blood pressure and their joint cumulative effect on cardiovascular disease risk: a longitudinal cohort study

**DOI:** 10.1186/s12933-023-02058-1

**Published:** 2023-11-28

**Authors:** Kuangyi Wu, Huancong Zheng, Weiqiang Wu, Guanzhi Chen, Zefeng Cai, Zhiwei Cai, Yulong Lan, Dan Wu, Shouling Wu, Youren Chen

**Affiliations:** 1 Department of Cardiology, Second Affiliated Hospital of Shantou University Medical College, 69 Dongxia North RD., Shantou, 515000 China; 2 Shantou University Medical College, Shantou, China; 3 Cardiac Arrhythmia Center, Fuwai Hospital, National Center for Cardiovascular Diseases, Chinese Academy of Medical Sciences and Peking Union Medical College, Beijing, China; 4 Department of Cardiology, Sun Yat-sen Memorial Hospital of Sun Yat-sen University, Guangzhou, China; 5 Department of Cardiology, Kailuan General Hospital, 57 Xinhua East RD., Tangshan, 063000 China

**Keywords:** Insulin resistance, Cohort study, Longitudinal, Hypertension, Temporal relationship

## Abstract

**Background:**

Concurrent insulin resistance and elevated blood pressure are commonly observed in cardiovascular disease (CVD) and have long been proposed to contribute to CVD. However, the temporal relationship between them and the effect of their cumulative co-exposure on future incident CVD remains unclear.

**Methods:**

Longitudinal analysis of data on 57,192 participants from a real-world, prospective cohort study (Kailuan Study) was performed to address the temporal relationship between Triglyceride-Glucose Index (TyG, calculated as ln [TG (mg/dL) × FBG (mg/dL)/2]) and blood pressure (BP) assessed by cross-lagged analyses in an approximately 4-year exposure period (2006/2007 to 2010/2011). After excluding 879 participants with known diabetes, 56,313 nonCVD participants were included for further analysis of the CVD outcome. Cox regression models were used to examine the hazard ratios (HRs) upon the cumulative TyG (CumTyG) and BP(CumBP) in the exposure period.

**Results:**

The standard regression coefficient from baseline TyG to follow-up systolic BP was 0.0142 (95% CI 0.0059–0.0226), which was greater than the standard regression coefficient from baseline systolic BP to follow-up TyG (− 0.0390; 95% CI − 0.0469 to − 0.0311). The same results were observed in the cross-lag between TyG and diastolic blood pressure [0.0271 (0.0185 to 0.0356) vs. − 0.0372 (− 0.0451 to − 0.0293)]. During a median follow-up of 9.98 years, 3981 CVD cases occurred. Significant interactions were observed between the median CumTyG (8.61) and CumSBP thresholds (130, 140 mmHg) (*P* = 0.0149), the median CumTyG (8.61) and CumDBP thresholds (80, 90 mmHg) (*P* = 0.0441). Compared to CumTyG < 8.61 and CumSBP < 130 mmHg, after adjusting for potential confounding factors, the HR gradually increased in the high co-exposure groups. The hazard ratios (HRs) and 95% confidence intervals (CIs) for Q2–Q6 were 1.39 (1.24, 1.57), 1.94 (1.69, 2.22), 2.40 (2.12, 2.71), 2.74 (2.43, 3.10), and 3.07 (2.74, 3.45). Additionally, the CVD risks in the co-exposure were more prominent in younger participants.

**Conclusions:**

These findings suggest that elevated TyG has a greater impact on future blood pressure changes than vice versa. Dual assessment and management of insulin resistance and blood pressure contribute to the prevention of CVD, especially in younger individuals.

**Supplementary Information:**

The online version contains supplementary material available at 10.1186/s12933-023-02058-1.

## Introduction

Cardiovascular disease (CVD) is one of the principal contributors to both global morbidity and mortality [1]. A comprehensive analysis conducted by Liu et al. regarding the burden of CVD in China revealed that between 1990 and 2016, the prevalence of CVD doubled, reaching 94 million cases [2]. In China, CVD is the foremost cause of death and premature mortality, accounting for 40% of all deaths in the Chinese population [3]. Therefore, it poses a significant public health challenge and imposes a substantial economic burden on affected individuals. Consequently, the early identification of individuals who are at risk of developing CVD is important for risk stratification and the management of the condition.

Hypertension has been firmly established as an important risk factor for CVD [4]. Furthermore, insulin resistance (IR) is frequently present alongside hypertension in patients with CVD [5]. It is well known that IR plays a pivotal role in the development of CVD through effects on systemic lipid metabolism, resulting in dyslipidemia and endothelial dysfunction. It can also be induced by aberrant insulin signaling, which contributes to the formation of atherosclerotic plaques [6]. Notably, IR and hypertension share a common molecular basis [7]. Insulin, through the activation of the phosphatidylinositol 3-kinase (PI3K) pathway, increases the production of endothelial nitric oxide, leading to vasodilation. However, in the state of IR, the activation of this pathway is impaired, and activation of the mitogen-activated protein kinase pathway is stimulated, which promotes vasoconstriction and therefore hypertension [8]. In addition, in patients with hypertension, the activation of the renin-angiotensin-aldosterone system and an upregulation of signaling through the mineralocorticoid receptor lead to greater production of reactive oxygen species and oxidative stress, which further exacerbates IR [9]. Although a substantial body of evidence underscores the urgent need to translate these intricate biological relationships into epidemiological evidence to inform primary prevention strategies for use in the general population with respect to CVD, there have been few studies of the temporal relationship between IR and hypertension.

The triglyceride (TG)-glucose (TyG) index is the logarithmic product of the circulating fasting TG and glucose concentrations, and has been demonstrated to represent a simple marker for the assessment of IR [10]. Previous research has shown a significant association between a high TyG index and a higher risk of cardiovascular events [11]. Although prior studies have separately explored the associations of TyG and BP with CVD, there have been few studies of their cumulative combined effects. Therefore, to delve deeper into the interrelationship between TyG and BP and their joint effect on the risk of CVD, we conducted a longitudinal study based on data from a prospective cohort study, the Kailuan study. We aimed to evaluate the risk of cardiovascular events occurring over a period of approximately 4 years, considering the cumulative effects of TyG (calculated as ln [TG (mg/dL) × FBG (mg/dL)/2]) and BP (systolic and diastolic); and to elucidate the temporal relationship between TyG and BP during the exposure period, by means of a path analysis.

## Methods

### Study participants

The Kailuan study (registration number Chi-CTR-TRNC-11,001,489) is a large-scale prospective cohort study that was initiated in 2006 and is still ongoing. It focuses on investigating the risk factors for CVD and related diseases in a community-based cohort and the implementation of appropriate interventions. Details of the research design and methods have been previously published [12, 13]. Starting from 2006, the Kailuan General Hospital and its 10 affiliated hospitals have conducted health examinations of both current and retired employees of the Kailuan Group every 2 years. In addition to routine follow-up assessments, the occurrence of adverse events, such as CVD, have been monitored annually. The study was approved by the Kailuan General Hospital Ethics Committee and is conducted in accordance with the principles of the Declaration of Helsinki. Written informed consent was obtained from all the participants.

**Fig. 1:**
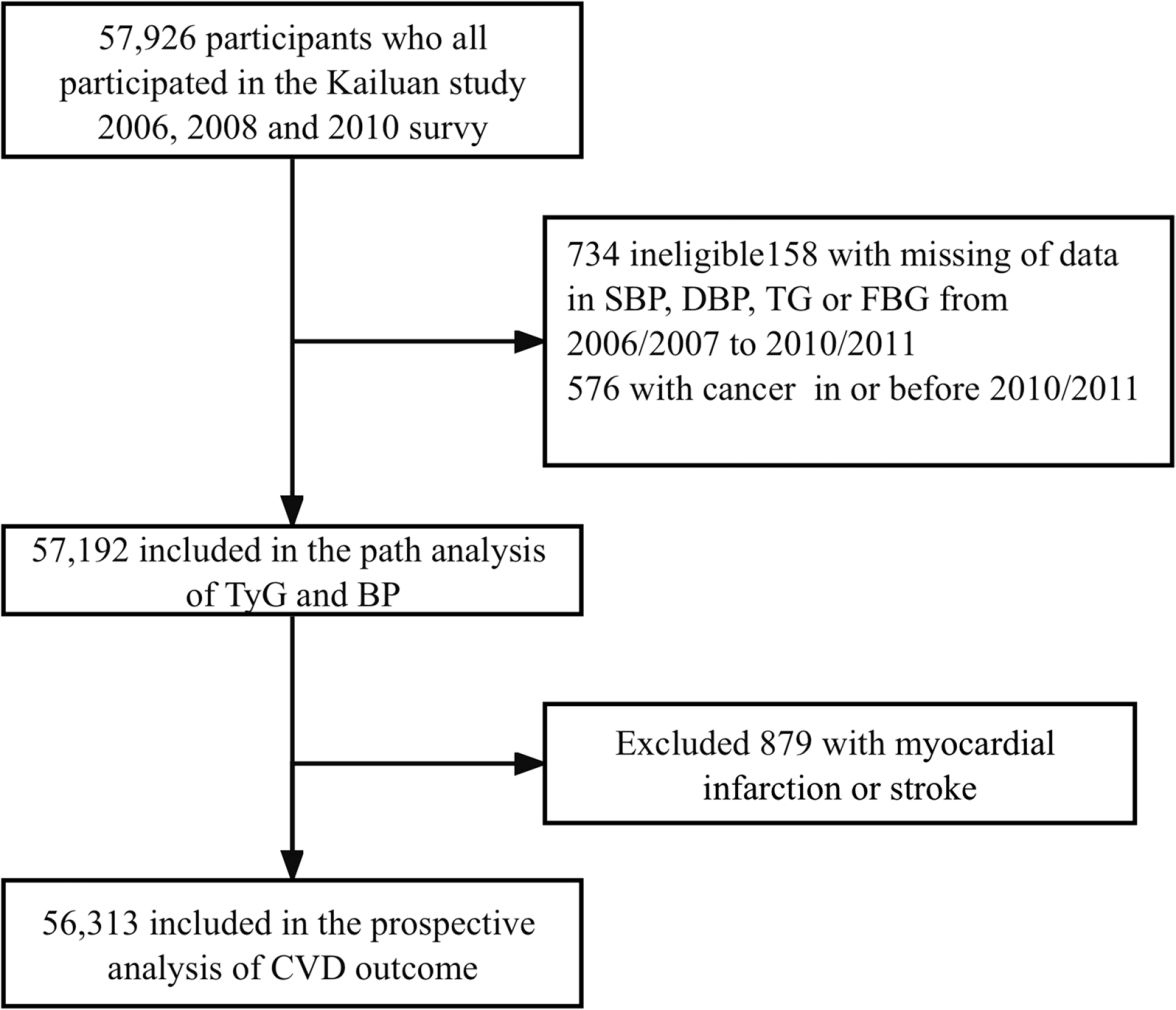
Flow chart of inclusion and exclusion in this study

Figure 1 illustrates the enrollment process for the participants in the present study. A total of 57,926 participants in the first three surveys of the Kailuan study (in 2006–2007, 2008–2009, and 2010–2011) were included. We excluded a total of 734 participants, including those with incomplete systolic BP (SBP), diastolic BP (DBP), TG, or fasting glucose (FG) data (n = 158), and those who had been diagnosed with cancer on or before the 2010–2011 survey (n = 576). Therefore, data for 57,192 participants were used in the path analysis. For the survival analysis, participants with a history of myocardial infarction or stroke before the start of follow-up (2010/2011) were also excluded (n = 879). Consequently, data for a total of 56,313 participants were included in the prospective analysis of CVD outcomes.

Information regarding the demographics, lifestyle, medical history, anthropometric measurements, and laboratory test results of the participants was collected at baseline and during follow-up surveys, as detailed elsewhere [14]. Briefly, trained personnel conducted questionnaire-based interviews to gather information regarding the age, sex, educational level, mean monthly income, smoking status, alcohol intake, physical activity, self-reported medical history (e.g., of hypertension and diabetes), and use of medication (e.g., hypoglycemic agents, antihypertensive drugs, and lipid-lowering medications) of the participants. Educational level was categorized as low (illiterate or elementary/middle school) or high (high school or college/university). A low income level was defined as a mean monthly income of ≤ 1000 yuan. Participants who were smoking at the time were defined as current smokers, and those who were consuming alcohol at the time were defined as current drinkers. The presence of hypertension was recorded on the basis of a self-reported history of hypertension, the use of antihypertensive medication, an SBP ≥ 140 mmHg, or a DBP ≥ 90 mmHg [15]. Diabetes was defined using an FBG ≥ 7.0 mmol/L, and/or a documented history of diabetes, and/or the use of antidiabetic medication [16]. Estimated glomerular filtration rate was calculated using the Chronic Kidney Disease Epidemiology Collaboration creatinine equation [17].

Trained medical professionals measured the height, body mass, SBP, and DBP of the participants. Their body mass index (BMI) was calculated as body mass (kg) divided by height (m) squared. Fasting blood samples were collected for analysis using a Hitachi 747 automated analyzer (Tokyo, Japan). The serum total cholesterol (TC), TG, HDL-C, low-density lipoprotein-cholesterol (LDL-C), fasting glucose (FG), creatinine, and high-sensitivity C-reactive protein (hs-CRP) concentrations were measured according to standard protocols. The TyG index was calculated as ln [TG (mg/dL) × FBG (mg/dL)/2].

### Assessment of exposures

**Fig. 2:**
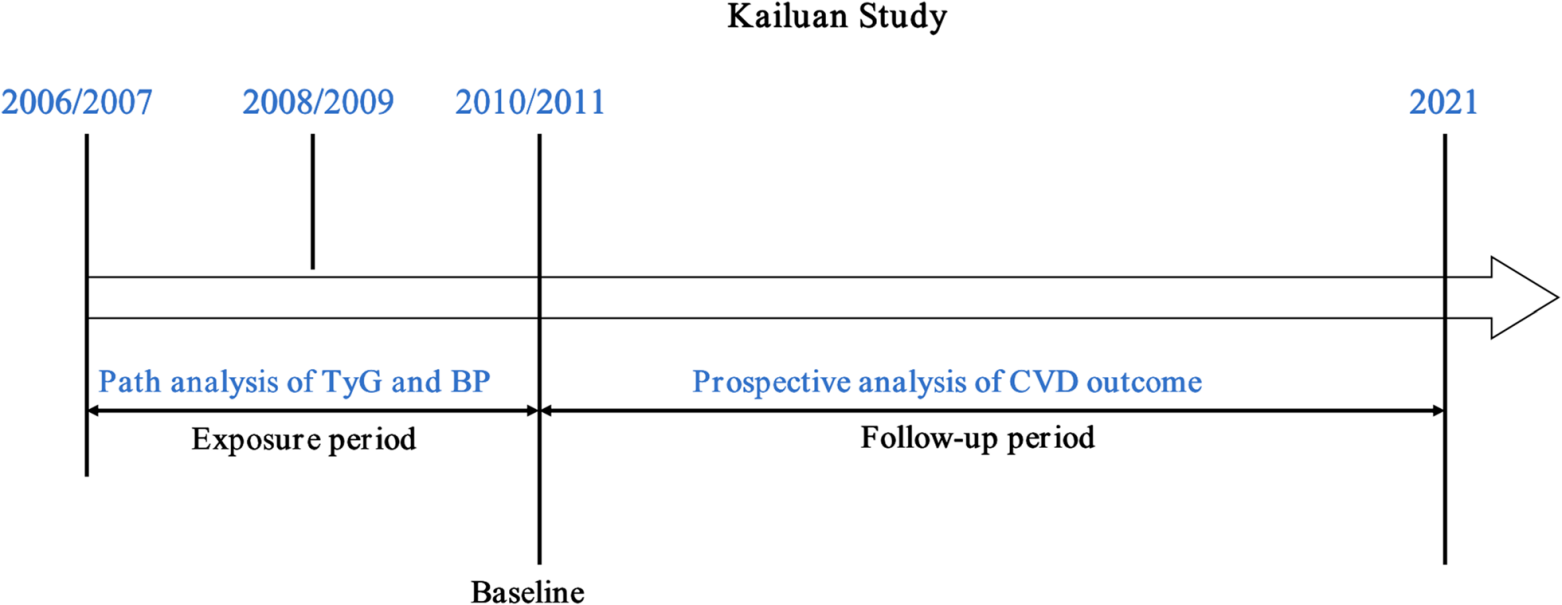
Strategies and design of the current study. The health examinations in the Kailuan Study were provided around every 2 years, except for the current last visit, with a time span of approximately 3 years owing to the influence of the COVID-19 pandemic. For the current study, the path analysis addressing the temporal relationship between TyG and BP was based on data measured in 2006/2007 and 2010/2011. For the survival analysis of CVD outcome, the cumulative exposure period was from 2006/2007 to 2010/2011. At the end of Visit_2010/2011, the participants were followed up through December 31, 2021. Baseline characteristics were based on the information in Visit_2010/2011

A brief summary of the study design and procedures is provided in Fig. 2. In the present cohort study, cumulative exposure was determined over a median period of 3.95 years (interquartile range: 3.73–4.29 years) preceding the follow-up period. The time-weighted cumulative TyG index (CumTyG) was calculated using the formula [(TyG_2006/2007 + TyG_2008/2009)/2 × (visit 1 − 2) + (TyG_2008/2009 + TyG_2010/2011)/2 × (visit 2 − 3)]/(visit 1 − 3); where TyG_2006/2007, TyG_2008/2009, and TyG_2009/2010 represent the TyG values calculated during the 2006/2007, 2008/2009, and 2010/2011 physical examinations, respectively; and visit 1 − 2 and visit 2 − 3 are the time intervals between the two named health surveys. The time-weighted cumulative SBP (CumSBP) and time-weighted cumulative DBP (CumDBP) were calculated using a similar approach. Participants were placed into groups on the basis of the median CumTyG value. Because no defined thresholds exist, and considering the high intra-individual variability over time, the suggested clinical cutoffs (< 130, 130–140, and ≥ 140 mmHg) for single SBP measurements in Asian populations were used as the CumSBP thresholds. Participants were also placed into three groups according to clinical thresholds of DBP (< 80, 80–90, and ≥ 90 mmHg) with respect to CumDBP.

### Ascertainment of outcomes

The primary outcome of the study was the incidence of CVD, defined as a composite of myocardial infarction and stroke. As previously described [18, 19], the participants’ records were linked to the Municipal Social Insurance Institution and the Hospital Discharge Register, which cover all the participants in the Kailuan study. In addition, discharge lists from the 11 hospitals between 2006 and 2021 were reviewed, and participants were interviewed biennially regarding their history of CVD. For suspected CVD events, three experienced physician adjudicators, who were blinded to the study design, reviewed the medical records of the participants. Myocardial infarction was defined using the ICD-10 code I21 and stroke was defined using the ICD-10 codes I63 or I60 to I61. Vital status information was obtained from the Hebei Provincial Vital Statistics Offices and, when necessary, by contacting members of the participants’ families, and was reviewed by physicians.

### Statistical analysis

We employed a standard cross-lagged panel design [20] to investigate the temporal relationship between TyG and BP during the exposure period, which had a median duration of 3.96 years, as illustrated in Additional file 1: Fig. S1. Taking SBP as an example, we simultaneously measured the path coefficients β1, representing the effect of TyG_2010/2011 on SBP_2006/2007, and β2, representing the effect of SBP_2010/2011 on TyG_2006/2007. These coefficients were adjusted for autoregressive effects. Significant path coefficients (β1 or β2) were taken to indicate the directionality of a relationship, and a significant difference between β1 and β2 was taken to be strong evidence for the direction of the relationship between the two variables over time. Statistically significant differences between β1 and β2 were identified using Student’s *t*-test. In this analysis, TyG and BP values were standardized, with a mean of 0 and a standard deviation (SD) of 1. The multivariable adjustment models used were as follows. Model 1 was adjusted for age, sex, smoking habits, alcohol consumption, BMI, and physical exercise status, all of which were assessed in 2010/2011. Model 2, building upon Model 1, was further corrected for heart rate, TC, hs-CRP, and the baseline use of antihypertensive, antidiabetic, or lipid-lowering medications.

For the baseline characteristics of participants in the prospective analysis of CVD risk, the median and interquartile range are used to summarize non-normally distributed continuous data, the mean ± standard deviation (SD) are used for normally distributed continuous data, and numerical values and percentages (%) are used for categorical data. To compare continuous datasets, we used the Kruskal–Wallis test or one-way analysis of variance (ANOVA). Kaplan–Meier method was used to the calculate cumulative CVD incidence for each group, and the log-rank test to compare the groups. Furthermore, using the proportional hazards assumption, with *P* > 0.05, we conducted likelihood ratio tests to evaluate the potential multiplicative interaction (INTm) between CumTyG and CumBP. Cox proportional hazards regression analysis was used to calculate HRs and 95% CIs for CVD in the six study groups. Model 1 was adjusted for sex, age at baseline; Model 2 was further adjusted for baseline heart rate, BMI, hs-CRP, HDL-C, and the use of antihypertensive, antidiabetic, or lipid-lowering medications at baseline; and Model 3 was further adjusted for smoking status (smoker or not), alcohol consumption (drinker or not), and substantial physical activity (yes or no).

Further INTm analyses were performed for the relationships between joint cumulative exposure, age, sex, and overweight status, and stratified analyses based on these covariates were performed according to the identified interactions. In addition, sensitivity analyses were performed to assess the robustness and consistency of the findings. First, to minimize the possibility for reverse causality, participants with outcome events within the first 2 years of follow-up were excluded. Second, because IR is associated with diabetes mellitus, and we repeated the survival analysis after excluding participants with diabetes mellitus at baseline. Third, we made an additional adjustment for the severity of fatty liver. Finally, to take into account the effect of medications on the outcome, we performed further survival analyses after excluding participants who were taking antihypertensive, hypoglycemic, or lipid-lowering medications during the follow-up period.

Statistical analyses were conducted in SAS software (version 9.4; SAS Institute, Cary, NC, USA), using the SAS Proc Calis procedure for the cross-lagged analysis. A two-sided *P*-value of < 0.05 was considered to represent statistical significance, except during the interaction testing, when a *P*-value of < 0.1 was accepted.

## Results

### Results of the cross-lagged analysis of the relationships of TyG with SBP and DBP

**Fig. 3:**
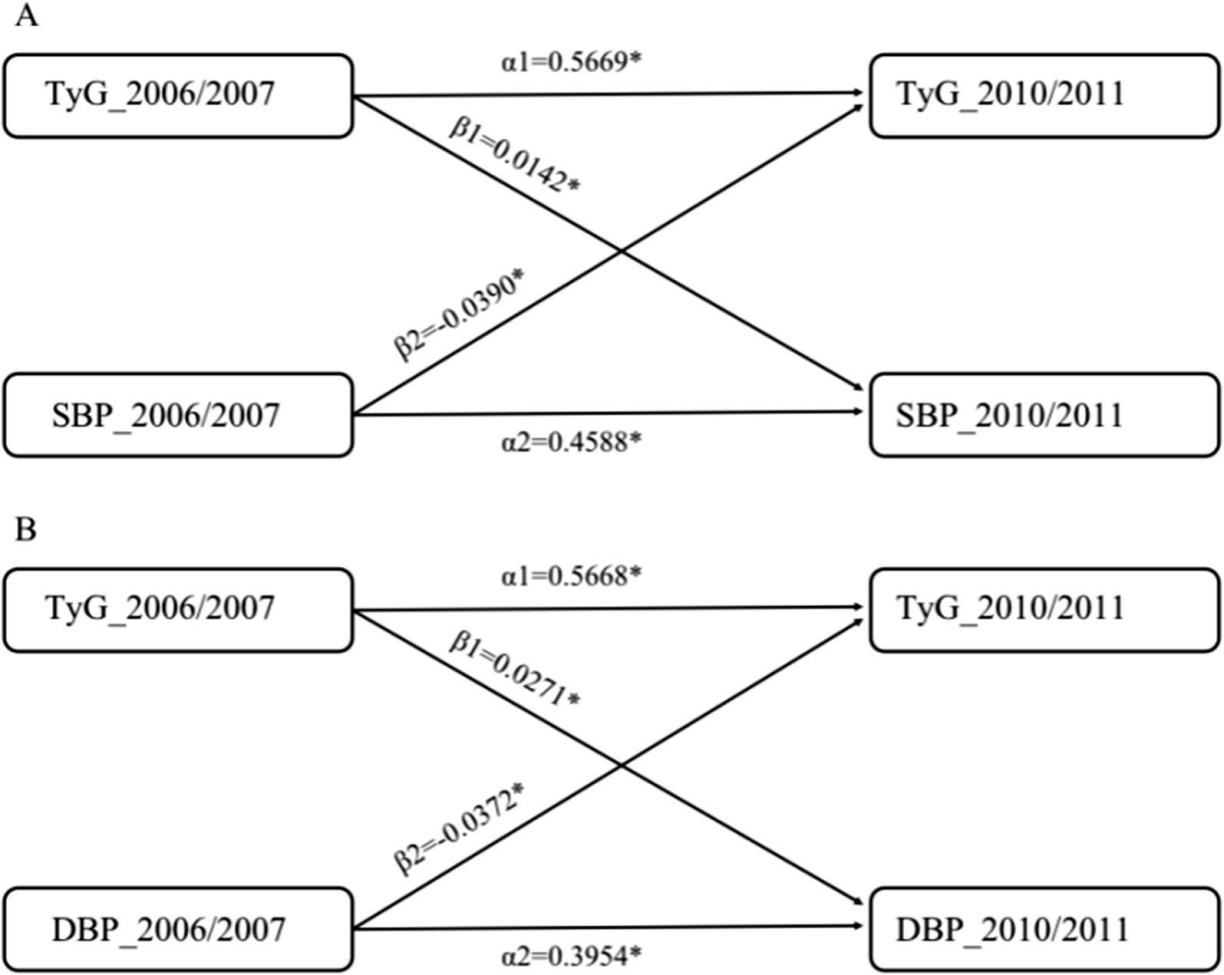
Cross-lagged standard regression coefficient of TyG and BP. *P < 0.001. **A** Cross-lagged standard regression coefficient of TyG and SBP. **B** Cross-lagged standard regression coefficient of TyG and DBP. The cross-lagged model was adjusted for age, gender, smoking habits, alcohol consumption, physical exercise, BMI, heart rate, TC, Hs-CRP, and baseline use of antihypertensive, antidiabetic, and lipid-lowering medications

A total of 57,192 participants who underwent repeated TyG and BP assessments were included in a standard cross-lagged panel design. Figure 3 and Additional file 1: Tables S1 and S2 depict the cross-lagged path analysis for TyG and BP. After adjustment for potential confounding factors, the path coefficient for baseline TyG to follow-up SBP (β1 = 0.014, 95% CI 0.006–0.023; *P* < 0.001) was significantly larger than that for baseline SBP to follow-up TyG (β2 = − 0.039, 95% CI − 0.045 to − 0.029; *P* < 0.001), with a *P*-value of < 0.0001 for the difference between β1 and β2. The proportions of variance for the follow-up TyG and SBP values (R^2^) that could be explained by the baseline TyG and SBP values were estimated to be 0.32 and 0.21, respectively (Additional file 1: Table S3). The path coefficient for baseline TyG to follow-up DBP (β1 = 0.040, 95% CI 0.033–0.048; *P* < 0.0001) was also significantly larger than that for baseline DBP to follow-up TyG (β2 = − 0.037, 95% CI − 0.045 to − 0.029; *P* = 0.001), with a *P*-value of < 0.0001 for the difference between β1 and β2. The proportions of variance for the follow-up TyG and DBP values (R^2^) that could be explained by the baseline TyG and DBP values were estimated to be 0.32 and 0.16, respectively (Additional file 1: Table S4).

### Baseline characteristics of the participants

At the beginning of the follow-up period, the baseline characteristics of the 56,313 participants who did not have CVD were evaluated (Table 1 and Additional file 1: Table S5). The participants were mostly male (43,066 [76.5%]) and had a mean (SD) age of 53.24 (11.92) at the start of the follow-up period. Participants with higher CumTyG and CumBP values at baseline had higher mean BMI, heart rate, and hs-CRP values; higher prevalences of hypertension and diabetes; and were more likely to be taking antihypertensive, antidiabetic, and lipid-lowering medications. Furthermore, those in the higher CumTyG and CumSBP strata were more likely to be male, current alcohol drinkers, and current smokers. Interestingly, increases in CumSBP were associated with increases in age. However, in strata where the CumSBP was ≥ 130 mmHg, increases in CumTyG were associated with decreases in age.

**Table 1:** Baseline characteristics of participants by cumulative TyG index and cumulative SBP

Characteristics	Total	G1	G2	G3	G4	G5	G6	P value
	(N = 56,313)	(N = 18,098)	(N = 13,309)	(N = 4632)	(N = 6239)	(N = 5426)	(N = 8609)	
Age, years	53.24 ± 11.92	49.27 ± 11.63	49.63 ± 10.69	56.86 ± 11.15	54.19 ± 10.55	62.13 ± 10.56	58.91 ± 10.38	< 0.01
Male (%)	43,066 (76.5)	11,804 (65.2)	10,565 (79.4)	3825.0 (82.6)	5251.0 (84.2)	4570.0 (84.2)	7051.0 (81.9)	< 0.01
CumTyG	8.66 ± 0.55	8.20 ± 0.29	9.05 ± 0.37	8.26 ± 0.26	9.12 ± 0.40	8.27 ± 0.25	9.14 ± 0.41	< 0.01
CumSBP, mmHg	129.35 ± 16.45	116.08 ± 8.61	119.34 ± 7.30	134.59 ± 2.88	134.77 ± 2.91	151.76 ± 9.31	151.82 ± 9.20	< 0.01
CumDBP, mmHg	83.54 ± 8.93	77.18 ± 6.12	79.83 ± 5.70	86.18 ± 5.73	87.37 ± 5.62	91.98 ± 7.66	93.11 ± 7.62	< 0.01
BMI, kg/m^2^	25.11 ± 3.37	23.63 ± 3.07	25.55 ± 3.09	24.84 ± 3.22	26.34 ± 3.20	25.21 ± 3.34	26.69 ± 3.38	< 0.01
eGFR, mL/min/1.73 m^2^	89.85 ± 18.55	94.12 ± 18.11	93.05 ± 17.92	87.20 ± 17.30	88.44 ± 18.02	81.71 ± 17.44	83.52 ± 18.34	< 0.01
Heart rate, beats/min	73.14 ± 9.85	71.60 ± 9.18	72.90 ± 9.37	72.80 ± 9.70	74.69 ± 10.08	73.65 ± 10.48	75.49 ± 10.74	< 0.01
FBG, mmol/L	5.59 ± 1.28	5.13 ± 0.69	5.74 ± 1.38	5.30 ± 0.77	6.08 ± 1.61	5.41 ± 0.88	6.27 ± 1.70	< 0.01
TC, mmol/L	4.97 ± 0.93	4.72 ± 0.87	5.12 ± 0.93	4.85 ± 0.88	5.18 ± 0.93	4.89 ± 0.90	5.24 ± 0.97	< 0.01
TG, mmol/L	1.29 (0.91–1.90)	0.96 (0.72–1.23)	1.82 (1.35–2.61)	0.99 (0.75–1.26)	1.80 (1.33–2.64)	0.97 (0.75–1.23)	1.78 (1.32–2.55)	< 0.01
HDL-C, mmol/L	1.55 ± 0.40	1.62 ± 0.41	1.47 ± 0.38	1.61 ± 0.41	1.47 ± 0.38	1.62 ± 0.41	1.46 ± 0.38	< 0.01
LDL-C, mmol/L	2.58 ± 0.77	2.43 ± 0.71	2.68 ± 0.74	2.52 ± 0.76	2.73 ± 0.77	2.50 ± 0.80	2.72 ± 0.83	< 0.01
HsCRP, mg/L	1.20 (0.63–2.70)	0.97 (0.50–2.10)	1.29 (0.70–2.67)	1.20 (0.62–2.70)	1.45 (0.76–3.00)	1.40 (0.76–3.20)	1.70 (0.81–3.40)	< 0.01
Physical activities (%)	8214.0 (14.6)	2411.0 (13.3)	1683.0 (12.6)	777.00 (16.8)	929.00 (14.9)	981.00 (18.1)	1433.0 (16.6)	< 0.01
Education (%)	15,605 (27.7)	6308.0 (34.9)	4632.0 (34.8)	868.00 (18.7)	1532.0 (24.6)	748.00 (13.8)	1517.0 (17.6)	< 0.01
Current drinker (%)	19,937 (35.4)	5606.0 (31.0)	5643.0 (42.4)	1560.0 (33.7)	2564.0 (41.1)	1554.0 (28.6)	3010.0 (35.0)	< 0.01
Current smokers (%)	21,641 (38.4)	6091.0 (33.7)	6006.0 (45.1)	1729.0 (37.3)	2780.0 (44.6)	1750.0 (32.3)	3285.0 (38.2)	< 0.01
Hypertension (%)	25,949 (46.1)	2864.0 (15.8)	3500.0 (26.3)	2870.0 (62.0)	4104.0 (65.8)	4813.0 (88.7)	7798.0 (90.6)	< 0.01
Diabetes mellitus (%)	8445.0 (15.0)	635.00 (3.51)	2323.0 (17.5)	302.00 (6.52)	1754.0 (28.1)	485.00 (8.94)	2946.0 (34.2)	< 0.01
Anti-hypertensives (%)	7305.0 (13.0)	333.00 (1.84)	607.00 (4.56)	561.00 (12.1)	995.00 (16.0)	1544.0 (28.5)	3265.0 (38.0)	< 0.01
Antidiabetic drugs (%)	2471.0 (4.39)	146.00 (0.81)	704.00 (5.29)	79.00 (1.71)	482.00 (7.73)	122.00 (2.25)	938.00 (10.9)	< 0.01
Lipid-lowering drugs (%)	548.00 (0.97)	50.00 (0.28)	142.00 (1.07)	19.00 (0.41)	89.00 (1.43)	49.00 (0.90)	199.00 (2.32)	< 0.01

G1: CumSBP < 130 mmHg and CumTyG < 8.61; G2: CumSBP < 130 mmHg and CumTyG ≥ 8.61; G3: 130 ≤ CumSBP < 140 mmHg and CumTyG < 8.61; G4: 130 ≤ CumSBP < 140 mmHg and CumTyG ≥ 8.61; G5: CumSBP ≥ 140 mmHg and CumTyG < 8.61; G6: CumSBP ≥ 140 mmHg and CumTyG ≥ 8.61

*BMI* body mass index, *CumTyG* cumulative triglyceride-glucose index, *CumSBP* cumulative systolic blood pressure, *CumDBP* cumulative diastolic blood pressure, *eGFR* estimated glomerular filtration rate, *FBG* fasting blood glucose, *HDL-C* high-density lipoprotein cholesterol, *HsCRP* high-sensitivity C-reactive protein, *LDL-C* low-density lipoprotein cholesterol, *TC* total cholesterol, *TG* triglyceride

### Results of the prospective study of the effect of a combination of CumTyG and CumSBP on the risk of CVD

Over a median follow-up period of 9.98 years (IQR: 9.51–10.30 years), 3981 CVD events were recorded among the 56,313 participants. The participants were then grouped on the basis of the median CumTyG value and the clinical thresholds of CumSBP (130 and 140 mmHg), and the incidences of CVD in the Q1, Q2, Q3, Q4, Q5, and Q6 groups were 2.96, 4.71, 8.30, 10.19, 14.69, and 15.85 per 1000 person-years, respectively. There was a significant interaction between CumTyG, categorized according to the median value, and CumSBP, categorized according to the clinical thresholds (*P*-INTm: 0.0149). There was also a significant interaction between CumTyG, categorized according to the median value, and CumDBP, categorized according to the clinical thresholds (80 and 90 mmHg) (*P*-INTm: 0.0441).

**Fig. 4:**
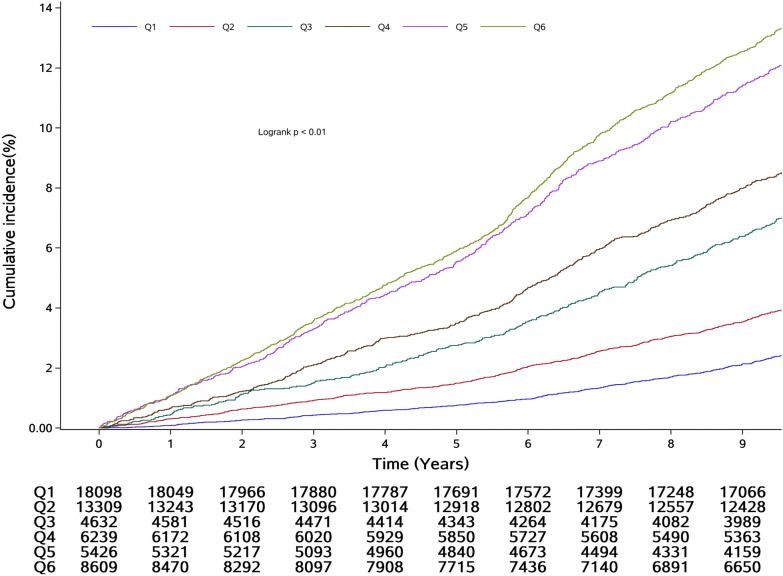
Kaplan–Meier incidence rate of CVD by cumulative TyG index and cumulative SBP

**Fig. 5:**
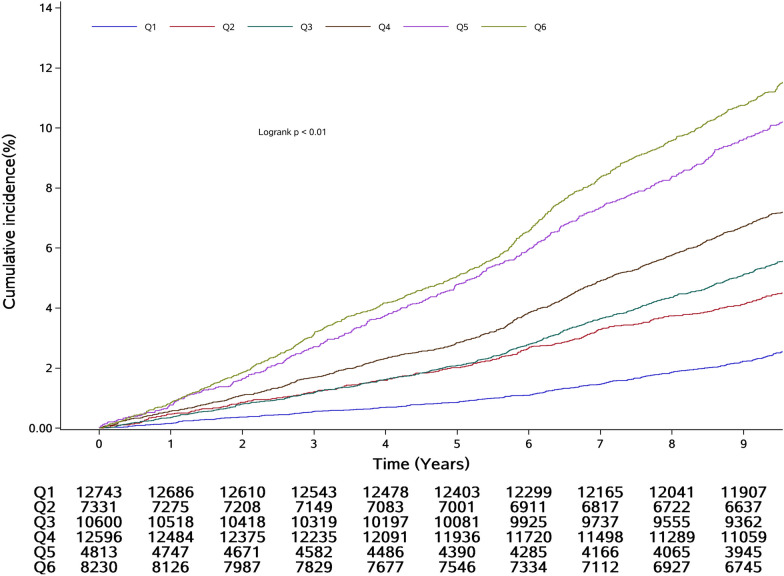
Kaplan–Meier incidence rate of CVD by cumulative TyG index and cumulative DBP. G1: CumDBP < 80 mmHg and CumTyG < 8.61; G2: CumDBP < 80 mmHg and CumTyG ≥ 8.61; G3: 80 ≤ CumDBP < 90 mmHg and CumTyG < 8.61; G4: 80 ≤ CumDBP < 90 mmHg and CumTyG ≥ 8.61; G5: CumDBP ≥ 90 mmHg and CumTyG < 8.61; G6: CumDBP ≥ 90 mmHg and CumTyG ≥ 8.61

Using Q1 (CumSBP < 130 mmHg and CumTyG < 8.61) as the reference group, and after adjustment for age, sex, heart rate, BMI, CRP, HDL-C, and the use of antihypertensive and lipid-lowering medications (Model 2), the calculated HRs were higher in the more severe co-exposure groups. The HRs and 95% CIs for groups Q2–Q6 were 1.39 (1.24, 1.57), 1.94 (1.69, 2.22), 2.40 (2.12, 2.71), 2.74 (2.43, 3.10), and 3.07 (2.74, 3.45), respectively. After further adjustment for lifestyle, including smoking, drinking, and physical activity habits, in Model 3, the associations were weaker, with HRs (95% CIs) for CumTyG and CumSBP Q2–Q6 of 1.40 (1.24, 1.57), 1.92 (1.68, 2.20), 2.38 (2.11, 2.69), 2.73 (2.41, 3.08), and 3.04 (2.71, 3.42), respectively (Table 2). Figures 4 and 5 display Kaplan–Meier curves of the cumulative incidence of CVD, and show that the incidence of CVD gradually increased from the Q1 to the Q6 group, and significantly differed among the groups (*P* < 0.01, log-rank test).

**Table 2:** Association between co-exposure to CumTyG and CumBP and CVD incidence

	Combination of CumBP and CumTyG, HRs (95% CIs)						
	CumSBP < 130 mmHg CumTyG < 8.61	CumSBP < 130 mmHg CumTyG ≥ 8.61	130 ≤ CumSBP < 140 mmHg CumTyG < 8.61	130 ≤ CumSBP < 140 mmHg CumTyG ≥ 8.61	CumSBP ≥ 140 mmHg CumTyG < 8.61	CumSBP ≥ 140 mmHg CumTyG ≥ 8.61	
Event/total	522/18,098	606/13,309	358/4632	592/6239	700/5426	1203/8609	
Incidence rate	2.96	4.71	8.30	10.19	14.69	15.85	
Unadjusted model	Reference	1.58 (1.40, 1.78)	2.86 (2.50, 3.27)	3.47 (3.09, 3.91)	5.20 (4.64, 5.82)	5.58 (5.03, 6.18)	
Model 1	Reference	1.52 (1.35, 1.71)	2.03 (1.78, 2.33)	2.77 (2.46, 3.12)	3.00 (2.67, 3.38)	3.75 (3.37, 4.16)	
Model 2	Reference	1.39 (1.24, 1.57)	1.94 (1.69, 2.22)	2.40 (2.12, 2.71)	2.74 (2.43, 3.10)	3.07 (2.74, 3.45)	
Model 3	Reference	1.40 (1.24, 1.57)	1.92 (1.68, 2.20)	2.38 (2.11, 2.69)	2.73 (2.41, 3.08)	3.04 (2.71, 3.42)	

	CumDBP < 80 mmHg CumTyG < 8.61	CumDBP < 80 mmHg CumTyG ≥ 8.61	80 ≤ CumDBP < 90 mmHg CumTyG < 8.61	80 ≤ CumDBP < 90 mmHg CumTyG ≥ 8.61	CumDBP ≥ 90 mmHg CumTyG < 8.61	CumDBP ≥ 90 mmHg CumTyG ≥ 8.61	
Event/Total	367/12,743	362/7331	676/10,600	1021/12,596	537/4813	1018/8230	
Incidence rate	2.98	5.21	6.75	8.61	12.28	13.61	
Unadjusted model	Reference	1.75 (1.51, 2.02)	2.27 (2.00, 2.57)	2.89 (2.56, 3.25)	4.16 (3.65, 4.76)	4.60 (4.08, 5.19)	
Model 1	Reference	1.59 (1.37, 1.84)	1.74 (1.53, 1.97)	2.43 (2.16, 2.74)	2.88 (2.52, 3.30)	3.65 (3.24, 4.12)	
Model 2	Reference	1.43 (1.23, 1.65)	1.65 (1.45, 1.87)	2.08 (1.83, 2.35)	2.55 (2.22, 2.93)	2.88 (2.53, 3.28)	
Model 3	Reference	1.42 (1.23, 1.64)	1.64 (1.44, 1.86)	2.07 (1.83, 2.34)	2.51 (2.19, 2.88)	2.85 (2.50, 3.24)	

Model 1: adjusted for age (continuous), gender(categorical)

Model 2: model 1 + heart rate, BMI (continuous), hs-CRP (continuous), HDL (continuous), antihypertensives (yes or no), lipid-lowering drugs (yes or no)

Model 3: model 2 + smoking status, drinking status, physical exercise

The incident rate is per 1000 person-years

*P* for interaction: CumTyG × CumSBP = 0.0149; *P* for interaction: CumTyG× CumDBP = 0.0441

In the stratified analyses, a significant interaction was found between co-exposure and age (*P*-INTm = 0.0001). Participants younger than 45 years of age were found to have a significantly higher risk of cardiovascular disease when they had IR and hypertension, with an HR (95% CI) of 4.35 (2.78, 6.82) compared to an HR (95% CI) of 2.40 (2.01, 2.85) for older participants. However, there was no statistically significant interaction between baseline sex and overweight status with respect to the incidence of CVD (Additional file 1: Tables S6 and S7).

Similar significant results were obtained in sensitivity analyses when participants with type 2 diabetes at baseline or who experienced a CVD event within the first 2 years of follow-up were excluded. However, there was a lower risk of a CVD event when the severity of fatty liver at baseline was also adjusted for. Furthermore, the results were consistent when participants who were taking antihypertensive drugs, hypoglycemic drugs, or lipid-lowering drugs were excluded (Additional file 1: Tables S8 and S9).

## Discussion

The findings of this community-based cohort study of 57,192 participants provide support for the existence of a bidirectional relationship between TyG and BP, with TyG having a stronger effect on subsequent BP than vice versa. Furthermore, in 56,313 participants without pre-existing CVD, we found a significant interaction between high BP and IR with respect to the risk of CVD. Specifically, the risk of CVD was significantly higher in the presence of exposure to both problems, especially in younger individuals (age < 60 years).

While the close association between TyG and BP has been previously established [21, 22], the nature of the temporal relationship between TyG and BP has not been fully elucidated. In the present study, we employed cross-lagged path analysis, a robust statistical method for the dissection of temporal relationships between correlated variables, to address the question of which defect develops first. This analysis showed that there is a bidirectional relationship of TyG with both SBP and DBP, even after adjustment for potential confounders, such as hs-CRP concentration and the use of antidiabetic medication [23]. It is well known that hypertension is associated with oxidative stress and inflammatory responses, leading to the production of specific cytokines and free radicals that disrupt insulin receptor signaling [24]. However, in addition, these substances affect the differentiation and metabolism of adipocytes [25], increase the release of free fatty acids [26], and thereby further exacerbate insulin resistance. The present findings suggest that a high TyG has a larger effect on subsequent BP than vice versa, which underscores the importance of IR in this relationship.

IR can lead to hypertension through several mechanisms, including renal sodium retention [27], activation of the sympathetic nervous system [28], greater peripheral and renal vascular resistance [29], endothelial dysfunction [30], and abnormalities in intracellular and extracellular ion transport [31]. Furthermore, there is growing evidence that TyG is an independent risk factor for hypertension [32, 33]. A cohort study of 4600 adults who did not have hypertension demonstrated a significant association between a high TyG index and a higher risk of incident hypertension in Chinese adults [22], which is to some extent consistent with the present findings. Interestingly, we found a negative association between SBP and subsequent TyG in the current study. We propose that multiple factors that affect IR, such as age-related metabolic and inflammatory factors [34], may underlie this observation. Cross-sectional analysis of the baseline data showed that increasing age was associated with increases in CumBP, but that CumTyG decreased with increasing CumBP stratum. Because aging is a major contributor to high blood pressure [35], age might mediate the negative association between BP and TyG. In summary, the present findings offer a novel perspective on the temporal relationship between hypertension and IR, and suggest that further exploration of the potential mechanism involved is necessary [7].

Furthermore, the results of the present study indicate that the cumulative exposure to a combination of BP and TyG is independently associated with the risk of CVD events, independent of conventional risk factors. Previous research has shown that before the onset of CVD, IR and high BP are related physiological defects. For the first time, the present study has provided an epidemiological link between CumTyG and CumBP, suggesting that pathways temporally connecting TyG to SBP and DBP may play a role in the development of CVD. IR and hypertension may predispose toward CVD through common mechanisms, including (1) endothelial dysfunction, featuring impaired regulation of vascular tone by nitric oxide [36], (2) the promotion of vascular stiffness and atherosclerosis [37], and (3) the induction of myocardial fibrosis and alterations in ion channels [38]. All of these possibilities suggest the necessity for a comprehensive assessment of these processes from an epidemiological perspective.

We also found that the risk of CVD associated with exposure to a combination of hypertension and IR varies according to the age of the individuals concerned, with those under 60 years of age being at a much higher relative risk of CVD when exposed to these risk factors than older individuals. This is consistent with previous findings that the excess risk of CVD associated with hypertension and IR diminishes with age [39–42]. In the CALIBER study of electronic health records, the relative risk associated with hypertension was found to decrease with age [39]. Similarly, data from the National Diabetes Services Scheme (NDSS) and the Swedish National Diabetes Register showed that the development of type 2 diabetes when young is associated with a higher risk of mortality [40, 41]. The present study has extended these findings by showing that exposure to a combination of hypertension and IR increases the risk of CVD in relatively young individuals. This implies that efforts aimed at the prevention of CVD should commence early in such individuals, to reduce their lifelong risk of CVD. Strict BP control, the use of statins, good glycemic control, and support for sustainable weight loss strategies are all feasible measures.

The present study had several strengths. First, it was a large-scale study conducted in a Chinese adult population. Other advantages include the rigorous research protocol, quality control measures, the large and representative sample, and the availability of data regarding many important covariates. Second, we collected TyG and BP data longitudinally from the participants before they developed CVD, allowing us to directly assess the long-term effect of the joint cumulative exposure to TyG and BP on CVD risk. Third, we adjusted for a number of confounding factors and conducted several sensitivity analyses. Fourth, we used path analysis to help clarify the nature of the relationship between IR and hypertension in the general population. This demonstrated a bidirectional relationship between TyG and BP at the epidemiological level.

The study also had some limitations. First, we did not assess the homeostatic model of assessment-insulin resistance (HOMA-IR) of the participants, because this is complex and expensive, and therefore not suitable for routine monitoring. In addition, the TyG index has been shown to closely correlate with euglycemic-hyperinsulinemic clamp data and the HOMA-IR, and serves as a simple surrogate index [10]. Second, we cannot draw definitive conclusions regarding the causal relationship between the joint cumulative exposure to TyG and BP and CVD risk in the general population, owing to the observational nature of the study. Furthermore, despite adjustment for potential confounding factors, we cannot rule out the possibility of residual or unassessed confounding. Lastly, this was a community-based cohort study of mainly Han Chinese individuals, limiting its generalizability.

## Conclusions

The present findings suggest that high TyG has a larger effect on subsequent BP than vice versa. Thus, cumulative IR modifies the CVD risk associated with hypertension. Therefore, the assessment and management of IR and BP may contribute to the prevention of CVD, especially in younger individuals.

### Supplementary Information

**Additional file 1: Table S1.** Multivariable adjusted cross-lagged standard regression coefficient of TyG and SBP (n = 57,192). **Table S2.** Multivariable adjusted cross-lagged standard regression coefficient of TyG and DBP (n = 57,192). **Table S3.** Pearson correlation coefficients between log-transformed SBP and TyG at baseline and follow-up in the total cohort, adjusted for covariates. **Table S4.** Pearson correlation coefficients between log-transformed DBP and TyG at baseline and follow-up in the total cohort, adjusted for covariates. **Table S5.** Baseline characteristics of participants by cumulative TyG index and cumulative DBP. **Table S6.** Stratified analysis between co-exposure to CumTyG and CumSBP and CVD incidence. **Table S7.** Stratified analysis between co-exposure to CumTyG and CumDBP and CVD incidence. **Table S8.** Sensitivity analyses of incidence of CVD with co-exposure stratified by CumSBP and CumTyG (median). **Table S9.** Sensitivity analyses of incidence of CVD with co-exposure stratified by CumDBP and CumTyG (median). **Figure S1.** Cross-lagged analysis design panel.

## Data Availability

The datasets used and/or analyzed during the present study are available from the corresponding author on reasonable request.

## Abbreviations

CVD: Cardiovascular disease
IR: Insulin resistance
CumTyG: Cumulative triglyceride-glucose index
CumSBP: Cumulative systolic blood pressure
CumDBP: Cumulative diastolic blood pressure
eGFR: Estimated glomerular filtration rate
FBG: Fasting blood glucose
HDL-C: High-density lipoprotein cholesterol
HsCRP: High-sensitivity C-reactive protein
LDL-C: Low-density lipoprotein cholesterol
TC: Total cholesterol
TG: Triglyceride
BMI: Body mass index
