# Supplementary material for: Temporal relationship between triglyceride-glucose index and blood pressure and their joint cumulative effect on cardiovascular disease risk: a longitudinal cohort study
Source: Cardiovasc Diabetol. 2023 Nov 28;22:332. doi: 10.1186/s12933-023-02058-1 (PMC10685547; doi:10.1186/s12933-023-02058-1)
Supplement: Supplementary file 1 — Additional file 1: Table S1. Multivariable adjusted cross-lagged standard regression coefficient of TyG and SBP (n = 57,192). Table S2. Multivariable adjusted cross-lagged standard regression coefficient of TyG and DBP (n = 57,192). Table S3. Pearson correlation coefficients between log-transformed SBP and TyG at baseline and follow-up in the total cohort, adjusted for covariates. Table S4. Pearson correlation coefficients between log-transformed DBP and TyG at baseline and follow-up in the total cohort, adjusted for covariates. Table S5. Baseline characteristics of participants by cumulative TyG index and cumulative DBP. Table S6. Stratified analysis between co-exposure to CumTyG and CumSBP and CVD incidence. Table S7. Stratified analysis between co-exposure to CumTyG and CumDBP and CVD incidence. Table S8. Sensitivity analyses of incidence of CVD with co-exposure stratified by CumSBP and CumTyG (median). Table S9. Sensitivity analyses of incidence of CVD with co-exposure stratified by CumDBP and CumTyG (median). Figure S1. Cross-lagged analysis design panel. [file 12933_2023_2058_MOESM1_ESM.docx]

**Additional file 1**

Content list:

Table S1. Multivariable adjusted cross-lagged standard regression coefficient of TyG and SBP (n=57,192).

Table S2. Multivariable adjusted cross-lagged standard regression coefficient of TyG and DBP (n=57,192).

Table S3. Pearson correlation coefficients between log-transformed SBP and TyG at baseline and follow-up in the total cohort, adjusted for covariates.

Table S4. Pearson correlation coefficients between log-transformed DBP and TyG at baseline and follow-up in the total cohort, adjusted for covariates.

Table S5. Baseline characteristics of participants by cumulative TyG index and cumulative DBP.

Table S6. Stratified analysis between co-exposure to CumTyG and CumSBP and CVD incidence.

Table S7. Stratified analysis between co-exposure to CumTyG and CumDBP and CVD incidence.

Table S8. Sensitivity analyses of incidence of CVD with co-exposure stratified by CumSBP and CumTyG (median).

Table S9. Sensitivity analyses of incidence of CVD with co-exposure stratified by CumDBP and CumTyG (median).

Figure S1. Cross-lagged analysis design panel.

# Table S1. Multivariable adjusted cross-lagged standard regression coefficient of TyG and SBP (n=57,192)

|  | **R^2^ of TyG** | **R^2^ of SBP** | **TyG_2006/2007 to TyG_2010/2011** | **SBP_2006/2007 to SBP_2010/2011** | **TyG_2006/2007 to SBP_2010/2011** | **SBP_2006/2007 to TyG_2010/2011** | ***P* value** |
| --- | --- | --- | --- | --- | --- | --- | --- |
| Crude model | 0.4267 | 0.4105 | 0.6559 (0.6506,0.6613) | 0.6221 (0.6165,0.6277) | 0.0631 (0.0555 to 0.0706) | -0.0112 (-0.0187 to -0.0038) | P<0.001 |
| Model 1 | 0.3683 | 0.2703 | 0.6087 (0.6030,0.6144) | 0.5136 (0.5070,0.5202) | 0.0314 (0.0233 to 0.0395) | -0.0113 (-0.0190 to -0.0037) | P<0.001 |
| Model 2 | 0.3163 | 0.2126 | 0.5669 (0.5608,0.5730) | 0.4588 (0.4517,0.4659) | 0.0142 (0.0059 to 0.0226) | -0.0390 (-0.0469 to -0.0311) | P<0.001 |

Model 1: adjusted for age (continuous), sex, education, smoking status, drinking status, physical activities, BMI (continuous);

Model 2: further adjusted for HR (continuous), TC (continuous), Hs-CRP (continuous), antihypertensives (yes or no), hypoglycemic agents (yes or no) and lipid-lowering drugs (yes or no) measured in 2010/2011.

Abbreviations: TyG, triglyceride-glucose index; SBP, systolic blood pressure; BMI, body mass index; HR, heart rate; TC, total cholesterol; Hs-CRP, high-sensitivity C-reactive protein.

# Table S2. Multivariable adjusted cross-lagged standard regression coefficient of TyG and DBP (n=57,192)

|  | **R^2^ of TyG** | **R^2^ of SBP** | **TyG_2006/2007 to TyG_2010/2011** | **DBP_2006/2007 to DBP_2010/2011** | **TyG_2006/2007 to DBP_2010/2011** | **DBP_2006/2007 to TyG_2010/2011** | ***P* value** |
| --- | --- | --- | --- | --- | --- | --- | --- |
| Crude model | 0.4266 | 0.2853 | 0.6523 (0.6469,0.6577) | 0.5045 (0.4977,0.5112) | 0.0892 (0.0811 to 0.0973) | 0.0032 (-0.0043 to 0.0107) | P<0.001 |
| Model 1 | 0.3684 | 0.2013 | 0.6095 (0.6038,0.6152) | 0.4403 (0.4331,0.4475) | 0.0395 (0.0311 to 0.0479) | -0.0166 (-0.0242 to -0.0089) | P<0.001 |
| Model 2 | 0.3162 | 0.1603 | 0.5668 (0.5607,0.5729) | 0.3954 (0.3878,0.4030) | 0.0271 (0.0185 to 0.0356) | -0.0372 (-0.0451 to -0.0293) | P<0.001 |

Model 1: adjusted for age (continuous), sex, education, smoking status, drinking status, physical activities, BMI (continuous);

Model 2: further adjusted for HR (continuous), TC (continuous), Hs-CRP (continuous), antihypertensives (yes or no), hypoglycemic agents (yes or no) and lipid-lowering drugs (yes or no) measured in 2010/2011 and the time intervals between examinations.

Abbreviations: TyG, triglyceride-glucose index; DBP, diastolic blood pressure; BMI, body mass index; HR, heart rate; TC, total cholesterol; Hs-CRP, high-sensitivity C-reactive protein.

# Table S3. Pearson correlation coefficients between log-transformed SBP and TyG at baseline and follow-up in the total cohort, adjusted for covariates

| **Variables** | **SBP_2006/2007** | **TyG_2006/2007** | **SBP_2010/2011** |
| --- | --- | --- | --- |
| TyG_2006/2007 | 0.2237 (0.2159, 0.2314) * | - | - |
| SBP_2010/2011 | 0.5740 (0.5685, 0.5794) * | 0.1959 (0.1880, 0.2037) * | - |
| TyG_2010/2011 | 0.1366 (0.1286, 0.1446) * | 0.5878 (0.5825, 0.5931) * | 0.1769 (0.1690, 0.1848) ^*^ |

Covariates included in the models were age (continuous), sex, education, smoking status, drinking status, physical activities, BMI (continuous), HR (continuous), TC (continuous), hs-CRP (continuous), antihypertensives (yes or no), hypoglycemic agents (yes or no) and lipid-lowering drugs (yes or no) measured in 2010/2011.

“^*^” indicates a two-tailed P < 0.001

# Table S4. Pearson correlation coefficients between log-transformed DBP and TyG at baseline and follow-up in the total cohort, adjusted for covariates

| **Variables** | **DBP_2006/2007** | **TyG_2006/2007** | **DBP_2010/2011** |
| --- | --- | --- | --- |
| TyG_2006/2007 | 0.2280 (0.2202, 0.2357) * | - | - |
| DBP_2010/2011 | 0.4744 (0.4680, 0.4807) * | 0.1953 (0.1874, 0.2031) * | - |
| TyG_2010/2011 | 0.1516 (0.1436, 0.1596) * | 0.5878 (0.5825, 0.5931) * | 0.2035 (0.1956, 0.2113) ^*^ |

Covariates included in the models were age (continuous), sex, education, smoking status, drinking status, physical activities, BMI (continuous), HR (continuous), TC (continuous), hs-CRP (continuous), antihypertensives (yes or no), hypoglycemic agents (yes or no) and lipid-lowering drugs (yes or no) measured in 2010/2011.

“^*^” indicates a two-tailed P < 0.001.

**Table S5: Baseline characteristics of participants by cumulative TyG index and cumulative DBP**

| **Characteristics** | **Total** | **G1** | **G2** | **G3** | **G4** | **G5** | **G6** | **P Value** |  |
| --- | --- | --- | --- | --- | --- | --- | --- | --- | --- |
|  |  |  |  |  |  |  |  |  |  |
|  | **(N=56,313)** | **(N=12,743)** | **(N=7,331)** | **(N=10,600)** | **(N=12,596)** | **(N=4,813)** | **(N=8,230)** |  |  |
| Age, years | 53.24±11.92 | 49.93±13.08 | 52.21±12.81 | 54.78±11.84 | 53.40±11.24 | 57.18±10.14 | 54.72±9.72 | <.01 |  |
| Male (%) | 43066 (76.5) | 7496.0 (58.8) | 5187.0 (70.8) | 8555.0 (80.7) | 10479 (83.2) | 4148.0 (86.2) | 7201.0 (87.5) | <.01 |  |
| CumTyG | 8.66±0.55 | 8.17±0.29 | 9.03±0.35 | 8.25±0.27 | 9.10±0.39 | 8.28±0.25 | 9.16±0.41 | <.01 |  |
| CumSBP, mmHg | 129.35±16.45 | 114.48±10.50 | 118.25±11.12 | 130.12±11.21 | 131.33±11.02 | 147.45±12.47 | 147.63±12.16 | <.01 |  |
| CumDBP, mmHg | 83.54±8.93 | 73.99±4.33 | 75.38±3.68 | 84.14±2.86 | 84.63±2.88 | 95.65±4.65 | 96.05±4.92 | <.01 |  |
| BMI, kg/m^2^ | 25.11±3.37 | 23.27±3.00 | 25.08±3.04 | 24.55±3.11 | 26.12±3.12 | 25.51±3.35 | 26.90±3.36 | <.01 |  |
| eGFR,  mL/min/1.73 m^2^ | 89.85±18.55 | 94.40±18.62 | 92.20±18.47 | 88.48±17.81 | 88.76±18.50 | 85.13±17.78 | 86.91±18.27 | <.01 |  |
| Heart rate, beats/min | 73.14±9.85 | 71.29±9.21 | 72.24±9.45 | 72.40±9.52 | 73.95±9.87 | 74.12±10.23 | 75.96±10.44 | <.01 |  |
| FBG, mmol/L | 5.59±1.28 | 5.12±0.71 | 5.80±1.49 | 5.26±0.77 | 5.96±1.52 | 5.33±0.77 | 6.15±1.64 | <.01 |  |
| TC, mmol/L | 4.97±0.93 | 4.71±0.87 | 5.14±0.96 | 4.81±0.87 | 5.16±0.94 | 4.86±0.89 | 5.20±0.95 | <.01 |  |
| TG, mmol/L | 1.59±1.05 | 1.00±0.44 | 2.07±1.12 | 1.06±0.48 | 2.14±1.18 | 1.07±0.44 | 2.23±1.22 | <.01 |  |
| HDL-C, mmol/L | 1.55±0.40 | 1.64±0.41 | 1.49±0.38 | 1.61±0.40 | 1.46±0.38 | 1.60±0.41 | 1.46±0.38 | <.01 |  |
| LDL-C, mmol/L | 2.58±0.77 | 2.40±0.72 | 2.69±0.76 | 2.49±0.75 | 2.71±0.77 | 2.53±0.77 | 2.71±0.80 | <.01 |  |
| HsCRP,mg/L | 1.20(0.63–2.70) | 0.90(0.50–2.00) | 1.24(0.70–2.61) | 1.17(0.60–2.61) | 1.40(0.70–2.96) | 1.30(0.70–3.07) | 1.60(0.80–3.25) | <.01 |  |
| Physical activities (%) | 8214.0 (14.6) | 1733.0 (13.6) | 1067.0 (14.6) | 1684.0 (15.9) | 1795.0 (14.3) | 752.00 (15.6) | 1183.0 (14.4) | <.01 |  |
| Education (%) | 15605 (27.7) | 4758.0 (37.3) | 2545.0 (34.7) | 2434.0 (23.0) | 3468.0 (27.5) | 732.00 (15.2) | 1668.0 (20.3) | <.01 |  |
| Current drinker (%) | 19937 (35.4) | 3439.0 (27.0) | 2585.0 (35.3) | 3641.0 (34.3) | 5138.0 (40.8) | 1640.0 (34.1) | 3494.0 (42.5) | <.01 |  |
| Current smokers (%) | 21641 (38.4) | 3822.0 (30.0) | 2936.0 (40.1) | 3944.0 (37.2) | 5488.0 (43.6) | 1804.0 (37.5) | 3647.0 (44.3) | <.01 |  |
| Hypertension (%) | 25949 (46.1) | 1365.0 (10.7) | 1327.0 (18.1) | 5025.0 (47.4) | 6742.0 (53.5) | 4157.0 (86.4) | 7333.0 (89.1) | <.01 |  |
| Diabetes mellitus (%) | 8445.0 (15.0) | 505.00 (3.96) | 1479.0 (20.2) | 581.00 (5.48) | 3111.0 (24.7) | 336.00 (6.98) | 2433.0 (29.6) | <.01 |  |
| Anti-hypertensives (%) | 7305.0 (13.0) | 240.00 (1.88) | 404.00 (5.51) | 889.00 (8.39) | 1638.0 (13.0) | 1309.0 (27.2) | 2825.0 (34.4) | <.01 |  |
| Antidiabetic drugs (%) | 2471.0 (4.39) | 132.00 (1.04) | 561.00 (7.65) | 150.00 (1.42) | 934.00 (7.42) | 65.00 (1.35) | 629.00 (7.64) | <.01 |  |
| Lipid-lowering drugs (%) | 548.00 (0.97) | 32.00 (0.25) | 81.00 (1.11) | 43.00 (0.41) | 189.00 (1.50) | 43.00 (0.89) | 160.00 (1.95) | <.01 |  |

G1: CumDBP<80mmHg and CumTyG<8.61; G2: CumDBP<80mmHg and CumTyG≥8.61; G3: 80≤CumDBP<90mmHg and CumTyG<8.61; G4:80≤CumDBP<90mmHg and CumTyG≥8.61; G5: CumDBP≥90mmHg and CumTyG<8.61; G6: CumDBP≥90mmHg and CumTyG≥8.61

*Abbreviations*: *BMI*, body mass index; *CumTyG*, cumulative triglyceride-glucose index; *CumSBP*, cumulative systolic blood pressure; *CumDBP*, cumulative diastolic blood pressure; *eGFR*, estimated glomerular filtration rate; *FBG*, fasting blood glucose; *HDL-C*, high-density lipoprotein cholesterol; *HsCRP*, high-sensitivity C-reactive protein; *LDL-C*, low-density lipoprotein cholesterol; *TC*, total cholesterol; *TG*, triglyceride

# Table S6. Stratified analysis between co-exposure to CumTyG and CumSBP and CVD incidence

|  | | **Combination of CumTyG and CumSBP, HRs (95% CIs)** | | | | | | | ***P* value for interaction** |
| --- | --- | --- | --- | --- | --- | --- | --- | --- | --- |
|  | CumSBP<130mmHg  CumTyG<8.66 | | | CumSBP<130mmHg  CumTyG≥8.66 | 130≤CumSBP<140mmHg  CumTyG<8.66 | 130≤CumSBP<140mmHg  CumTyG≥8.66 | CumSBP≥140mmHg  CumTyG<8.66 | CumSBP≥140mmHg  CumTyG≥8.66 |  |
| **Stratified by age** | | | | | | | | |  |
| **Age≥60** | | | | | | | | | 0.0001 |
| Event/Total | 200/2933 | | | 176/1992 | 177/1683 | 205/1651 | 443/3053 | 597/3760 |  |
| Incidence rate | 7.50 | | | 9.83 | 12.18 | 14.43 | 17.41 | 19.12 |  |
| Model 3 | Reference | | | 1.30(1.06,1.60) | 1.60(1.31,1.96) | 1.89(1.54,2.31) | 2.23(1.88,2.65) | 2.40(2.01,2.85) |  |
| **45≤Age<60** | | | | | | | | |  |
| Event/Total | 267/8973 | | | 367/7206 | 157/2387 | 337/3517 | 244/2120 | 555/4197 |  |
| Incidence rate | 2.15 | | | 3.88 | 6.33 | 8.82 | 11.57 | 13.57 |  |
| Model 3 | Reference | | | 1.46(1.25,1.72) | 1.96(1.61,2.39) | 2.57(2.17,3.03) | 3.44(2.87,4.12) | 3.54(3.01,4.12) |  |
| **Age<45** |  | |  |  |  |  |  |  |  |
| Event/Total | 55/6192 | | | 63/4111 | 24/562 | 50/1071 | 13/253 | 51/652 |  |
| Incidence rate | 0.89 | | | 1.54 | 4.31 | 4.75 | 5.21 | 8.10 |  |
| Model 3 | Reference | | | 1.25(0.86,1.83) | 3.14(1.92,5.15) | 3.09(2.03,4.70) | 3.51(1.88,6.56) | 4.35(2.78,6.82) |  |
| **Stratified by sex** | | | | |  |  |  |  |  |
| **Male** |  | | |  |  |  |  |  |  |
| Event/Total | 439/11804 | | | 516/10565 | 312/3825 | 526/5251 | 635/4570 | 1050/7051 |  |
| Incidence rate | 3.84 | | | 5.04 | 8.82 | 10.78 | 16.02 | 17.05 |  |
| Model 3 | Reference | | | 1.34(1.17,1.52) | 1.81(1.57,2.10) | 2.33(2.04,2.66) | 2.66(2.34,3.03) | 2.99(2.64,3.38) |  |
| **Female** |  | | |  |  |  |  |  | 0.3338 |
| Event/Total | 75/5247 | | | 219/2412 | 75/2045 | 143/2117 | 44/665 | 83/1029 |  |
| Incidence rate | 1.34 | | | 3.40 | 5.94 | 7.10 | 8.10 | 10.70 |  |
| Model 3 | Reference | | | 1.44(1.23,1.68) | 2.07(1.72,2.48) | 2.51(2.14,2.94) | 2.97(2.52,3.50) | 3.15(2.71,3.67) |  |
| **Stratified by BMI** | | | | | |  |  |  |  |
| **BMI**≥24 |  | | |  |  |  |  |  |  |
| Event/Total | 243/7726 | | | 430/9178 | 218/2722 | 466/4827 | 449/3426 | 952/6888 |  |
| Incidence rate | 3.22 | | | 4.84 | 8.56 | 10.35 | 14.84 | 15.61 |  |
| Model 3 | Reference | | | 1.34 (1.16,1.53) | 1.38 (1.19,1.61) | 1.63 (1.42,1.87) | 1.74 (1.54,1.98) | 1.95 (1.70,2.24) |  |
| **BMI<24** |  | | |  |  |  |  |  | 0.3820 |
| Event/Total | 279/10372 | | | 176/4131 | 140/1910 | 126/1412 | 251/2000 | 251/1721 |  |
| Incidence rate | 2.77 | | | 4.42 | 7.93 | 9.64 | 14.42 | 16.83 |  |
| Model 3 | Reference | | | 1.31(1.08,1.59) | 1.87(1.52,2.30) | 2.22(1.79,2.77) | 2.68(2.22,3.23) | 3.20(2.64,3.87) |  |

Model 3: adjusted for age (continuous), gender(categorical), heart rate, BMI (continuous), Hs-CRP (continuous), HDL (continuous), antihypertensives (yes or no), lipid-lowering drugs (yes or no), smoking status, drinking status, physical exercise.

The incident rate is per 1,000 person-years.

*Abbreviations*: *BMI*, body mass index; *CumTyG*, cumulative triglyceride-glucose index; *CumSBP*, cumulative systolic blood pressure; *CumDBP*, cumulative diastolic blood pressure; *eGFR*, estimated glomerular filtration rate; *FBG*, fasting blood glucose; *HDL-C*, high-density lipoprotein cholesterol; *HsCRP*, high-sensitivity C-reactive protein; *LDL-C*, low-density lipoprotein cholesterol; *TC*, total cholesterol; *TG*, triglyceride

#

# Table S7. Stratified analysis between co-exposure to CumTyG and CumDBP and CVD incidence

|  | | **Combination of CumTyG and CumDBP, HRs (95% CIs)** | | | | | | ***P* value for interaction** |
| --- | --- | --- | --- | --- | --- | --- | --- | --- |
|  | CumDBP<80mmHg  CumTyG<8.61 | | CumDBP<80mmHg  CumTyG≥8.61 | 80≤CumDBP<90mmHg  CumTyG<8.61 | 80≤CumDBP<90mmHg  CumTyG≥8.61 | CumDBP≥90mmHg  CumTyG<8.61 | CumDBP≥90mmHg  CumTyG≥8.61 |  |
| **Stratified by age** | | | | | | | |  |
| **Age≥60** | | | | | | | | 0.0001 |
| Event/Total | 204/2588 | | 174/1878 | 362/3300 | 453/3306 | 254/1781 | 351/2219 |  |
| Incidence rate | 8.91 | | 10.69 | 12.59 | 15.91 | 16.94 | 18.89 |  |
| Model 3 | Reference | | 1.19(0.97,1.46) | 1.34(1.13,1.60) | 1.65(1.39,1.96) | 1.69(1.39,2.04) | 1.78(1.47,2.14) |  |
| **45≤Age<60** | | | | | | | |  |
| Event/Total | 135/5605 | | 157/3385 | 281/5336 | 501/6672 | 252/2539 | 601/4863 |  |
| Incidence rate | 2.46 | | 4.80 | 5.43 | 7.82 | 10.55 | 13.35 |  |
| Model 3 | Reference | | 1.66(1.31,2.09) | 1.87(1.52,2.30) | 2.36(1.94,2.88) | 3.15(2.54,3.92) | 3.56(2.91,4.36) |  |
| **Age<45** |  | |  |  |  |  |  |  |
| Event/Total | 28/4550 | | 31/2068 | 33/1964 | 67/2618 | 31/493 | 66/1148 |  |
| Incidence rate | 0.62 | | 1.52 | 1.68 | 2.58 | 6.40 | 5.90 |  |
| Model 3 | Reference | | 1.78(1.05,3.01) | 1.86(1.11,3.13) | 2.45(1.52,3.95) | 5.50(3.19,9.47) | 4.14(2.48,6.92) |  |
| **Stratified by sex** | | | |  |  |  |  |  |
| **Male** |  | |  |  |  |  |  |  |
| Event/Total | 292/7496 | | 279/5187 | 601/8555 | 878/10479 | 493/4148 | 935/7201 |  |
| Incidence rate | 4.08 | | 5.68 | 7.49 | 8.92 | 13.17 | 14.35 |  |
| Model 3 | Reference | | 1.38(1.17,1.63) | 1.61(1.40,1.86) | 2.02(1.76,2.32) | 2.48(2.14,2.88) | 2.86(2.49,3.30) |  |
| **Female** |  | |  |  |  |  |  | 0.5785 |
| Event/Total | 75/5247 | | 219/2412 | 75/2045 | 143/2117 | 44/665 | 83/1029 |  |
| Incidence rate | 1.46 | | 4.08 | 3.77 | 7.13 | 6.98 | 8.61 |  |
| Model 3 | Reference | | 1.44(1.03,2.00) | 1.73(1.25,2.39) | 2.17(1.60,2.95) | 2.53(1.71,3.75) | 2.38(1.67,3.40) |  |
| **Stratified by BMI** | | | | |  |  |  |  |
| **BMI**≥24 |  | |  |  |  |  |  |  |
| Event/Total | 156/4819 | | 233/4636 | 389/5829 | 785/9523 | 365/3226 | 830/6734 |  |
| Incidence rate | 3.35 | | 5.30 | 7.05 | 8.76 | 12.45 | 13.52 |  |
| Model 3 | Reference | | 1.40(1.14,1.71) | 1.74(1.44,2.09) | 2.16(1.81,2.57) | 2.71(2.24,3.28) | 3.00(2.52,3.59) |  |
| **BMI<24** |  | |  |  |  |  |  | 0.7770 |
| Event/Total | 108/2733 | | 219/2412 | 126/1389 | 86/956 | 326/1853 | 278/1399 |  |
| Incidence rate | 2.76 | | 5.06 | 6.38 | 8.15 | 11.94 | 14.02 |  |
| Model 3 | Reference | | 1.36(1.09,1.71) | 1.67(1.39,2.00) | 2.03(1.68,2.46) | 2.58(2.09,3.19) | 3.09(2.50,3.82) |  |

Model 3: adjusted for age (continuous), gender(categorical), heart rate, BMI (continuous), hs-CRP (continuous), HDL (continuous), antihypertensives (yes or no), lipid-lowering drugs (yes or no), smoking status, drinking status, physical exercise.

The incident rate is per 1,000 person-years.

Abbreviations: BMI, body mass index; CumTyG, cumulative atherogenic index of plasma; CumCRP, cumulative high-sensitivity C-reactive protein; eGFR, estimated glomerular filtration rate; FBG, fasting blood glucose; HR, hazard ratio; TC, total cholesterol.

# Table S8. Sensitivity analyses of incidence of CVD with co-exposure stratified by CumSBP and CumTyG (median)

|  | **Combination of CumSBP and CumTyG, HRs (95% CIs)** | | | | | |
| --- | --- | --- | --- | --- | --- | --- |
|  | CumSBP<130mmHg  CumTyG<8.61 | CumSBP<130mmHg  CumTyG≥8.61 | 130≤CumSBP<140mmHg  CumTyG<8.61 | 130≤CumSBP<140mmHg  CumTyG≥8.61 | CumSBP≥140mmHg  CumTyG<8.61 | CumSBP≥140mmHg  CumTyG≥8.61 |
| **Excluded CVD events within 2 years of follow-up (3406/55649)** | | | | | | |
| Model 3 | Reference | 1.31(1.16,1.49) | 1.84(1.59,2.13) | 2.31(2.03,2.63) | 2.64(2.32,3.01) | 2.92(2.58,3.30) |
| **Excluded baseline diabetes (3127/49325)** | | | | | | |
| Model 3 | Reference | 1.31(1.15,1.49) | 1.93(1.68,2.22) | 2.21(1.93,2.54) | 2.69(2.37,3.06) | 2.85(2.52,3.24) |
| **Adjusted for** **baseline fatty liver (categorical) (3981/56313)** | | | | | | |
| Model 3 | Reference | 1.31(1.15,1.49) | 1.37 (1.19,1.58) | 1.57 (1.40,1.77) | 1.81 (1.63,2.02) | 1.98 (1.75,2.24) |
| **Excluded participants who used antihypertensive drugs (3035/48913)** | | | | | | |
| Model 3 | Reference | 1.38(1.22,1.56) | 1.88(1.63,2.17) | 2.45(2.16,2.79) | 2.65(2.32,3.02) | 3.04(2.69,3.44) |
| **Excluded participants who used hypoglycaemic drugs (3639/53755)** | | | | | | |
| Model 3 | Reference | 1.36(1.21,1.54) | 1.89(1.65,2.18) | 2.36(2.08,2.68) | 2.72(2.40,3.08) | 3.11(2.77,3.50) |
| **Excluded participants who used lipid-lowering drugs (3912/55662)** | | | | | | |
| Model 3 | Reference | 1.40(1.24,1.57) | 1.92(1.68,2.21) | 2.40(2.12,2.72) | 2.73(2.41,3.08) | 3.06(2.72,3.43) |

Model 3: adjusted for age (continuous), gender(categorical), heart rate, BMI (continuous), Hs-CRP (continuous), HDL (continuous), antihypertensives (yes or no), lipid-lowering drugs (yes or no), smoking status, drinking status, physical exercise.

Abbreviations: BMI, body mass index; CIs, confidence intervals; CumTyG, cumulative atherogenic index of plasma; CumCRP, cumulative high-sensitivity C-reactive protein; CVD, cardiovascular diseases; eGFR, estimated glomerular filtration rate; FBG, fasting blood glucose; HRs, hazard ratios; INTm, multiplicative interaction; SD, standard deviation; TC, total cholesterol.

# Table S9. Sensitivity analyses of incidence of CVD with co-exposure stratified by CumDBP and CumTyG (median)

|  | **Combination of CumDBP and CumTyG, HRs (95% CIs)** | | | | | |
| --- | --- | --- | --- | --- | --- | --- |
|  | CumDBP<80mmHg  CumTyG<8.61 | CumDBP<80mmHg  CumTyG≥8.61 | 80≤CumDBP<90mmHg  CumTyG<8.61 | 80≤CumDBP<90mmHg  CumTyG≥8.61 | CumDBP≥90mmHg  CumTyG<8.61 | CumDBP≥90mmHg  CumTyG≥8.61 |
| **Excluded CVD events within 2 years of follow-up (3406/55649)** | | | | | | |
| Model 3 | Reference | 1.35(1.15,1.58) | 1.65(1.44,1.89) | 2.05(1.79,2.34) | 2.50(2.16,2.90) | 2.81(2.44,3.23) |
| **Excluded baseline diabetes (3127/49325)** | | | | | | |
| Model 3 | Reference | 1.33(1.13,1.57) | 1.67(1.46,1.91) | 1.93(1.69,2.21) | 2.51(2.18,2.90) | 2.74(2.37,3.15) |
| **Adjusted for baseline fatty liver (categorical) (3981/56313)** | | | | | | |
| Model 3 | Reference | 1.39(1.20,1.61) | 1.63(1.44,1.86) | 2.01(1.78,2.28) | 2.49(2.17,2.86) | 2.76(2.42,3.15) |
| **Excluded participants who used antihypertensive drugs (3035/48913)** | | | | | | |
| Model 3 | Reference | 1.39(1.19,1.62) | 1.56(1.36,1.79) | 2.08(1.83,2.38) | 2.65(2.29,3.08) | 3.04(2.64,3.50) |
| **Excluded participants who used hypoglycaemic drugs (3639/53755)** | | | | | | |
| Model 3 | Reference | 1.38(1.19,1.62) | 1.62(1.42,1.84) | 2.06(1.82,2.34) | 2.50(2.17,2.87) | 2.90(2.54,3.32) |
| **Excluded participants who used lipid-lowering drugs (3912/55662)** | | | | | | |
| Model 3 | Reference | 1.41(1.21,1.63) | 1.63(1.43,1.85) | 2.07(1.83,2.35) | 2.50(2.18,2.87) | 2.85(2.50,3.25) |

Model 3: adjusted for age (continuous), gender(categorical), heart rate, BMI (continuous), Hs-CRP (continuous), HDL (continuous), antihypertensives (yes or no), lipid-lowering drugs (yes or no), smoking status, drinking status, physical exercise.

Abbreviations: BMI, body mass index; CIs, confidence intervals; CumTyG, cumulative atherogenic index of plasma; CumCRP, cumulative high-sensitivity C-reactive protein; CVD, cardiovascular diseases; eGFR, estimated glomerular filtration rate; FBG, fasting blood glucose; HRs, hazard ratios; INTm, multiplicative interaction; SD, standard deviation; TC, total cholesterol.

#

#
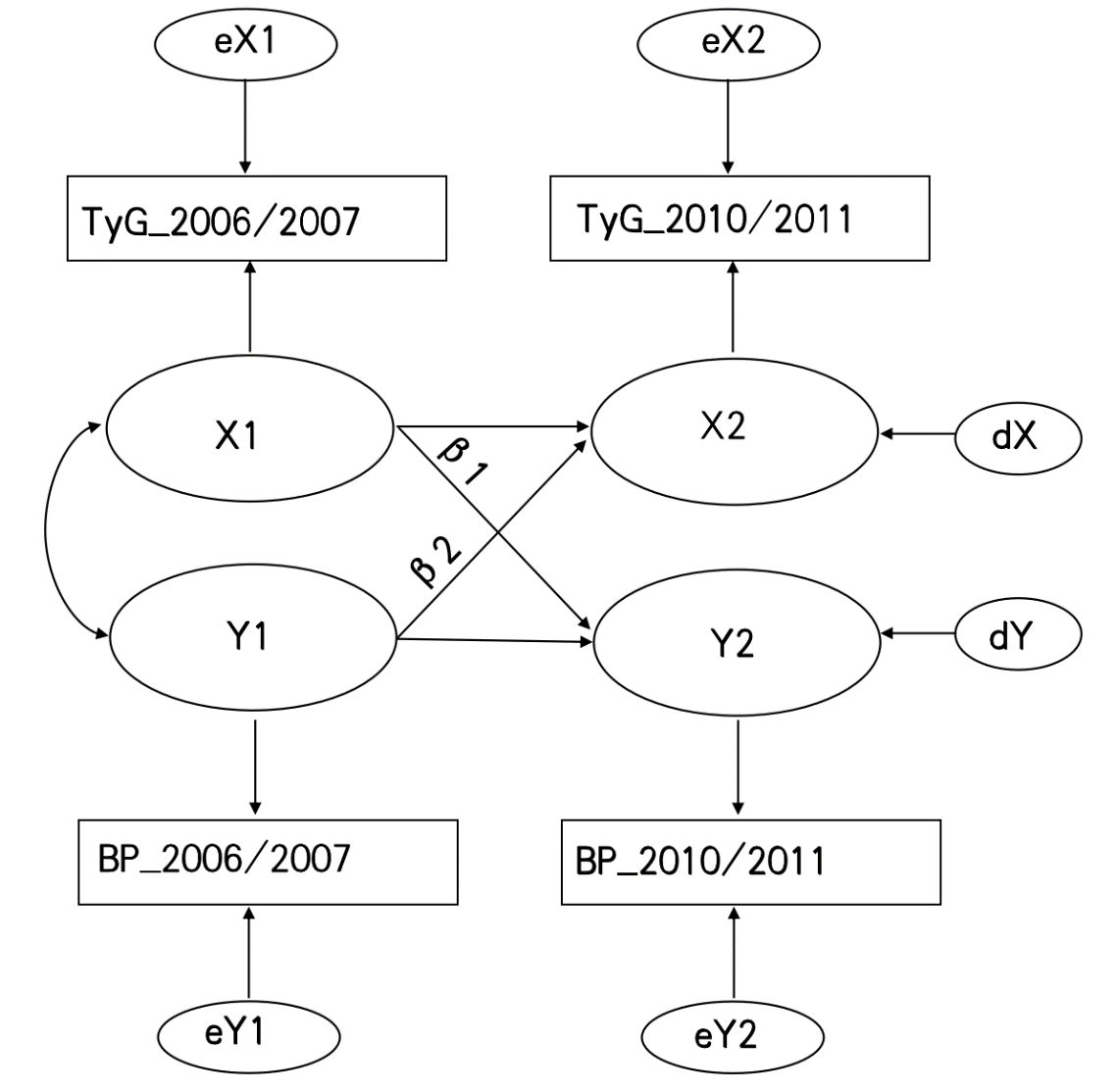


# Fig. S1. Cross-lagged analysis design panel

This design measured the effect size of BP measured at 2006/2007 on subsequent TyG measured at 2010/2011 (β1) and the effect size of TyG measured at 2006/2007 on subsequent BP measured at 2010/2011 (β2) simultaneously, adjusting for the auto-regressive effects.

Abbreviations: SBP, blood pressure; TyG, triglyceride-glucose index.
